# Simultaneous Measurements of Chlorophyll Concentration by Lidar, Fluorometry, above-Water Radiometry, and Ocean Color MODIS Images in the Southwestern Atlantic

**DOI:** 10.3390/s90100528

**Published:** 2009-01-16

**Authors:** Milton Kampel, João A. Lorenzzetti, Cristina M. Bentz, Raul A. Nunes, Rodolfo Paranhos, Frederico M. Rudorff, Alexandre T. Politano

**Affiliations:** 1 Instituto Nacional de Pesquisas Espaciais (INPE), PO Box 515, 12201-970, São José dos Campos, SP, Brazil; E-mails: {loren, fmr}@dsr.inpe.br; 2 PETROBRAS/CENPES, Cidade Universitária, Q.7, Ilha do Fundão, 21949-900, Rio de Janeiro, RJ, Brazil; E-mails: {cris, politano.gorceix}@petrobras.com.br; 3 Pontifícia Universidade Católica do Rio de Janeiro (PUC-Rio), PO Box 38008, 22453-900, Rio de Janeiro, RJ, Brazil; E-mail: nunes@dcmm.puc-rio.br; 4 Universidade Federal do Rio de Janeiro (UFRJ), Cidade Universitária, Av. Pau Brasil 211, Ilha do Fundão, 21941-590, Rio de Janeiro, RJ, Brazil; E-mail: rodpar@biologia.ufrj.br

**Keywords:** Chlorophyll, Lidar, MODIS, Above-water radiometry, Fluorometry

## Abstract

Comparisons between *in situ* measurements of surface chlorophyll-*a* concentration (CHL) and ocean color remote sensing estimates were conducted during an oceanographic cruise on the Brazilian Southeastern continental shelf and slope, Southwestern South Atlantic. *In situ* values were based on fluorometry, above-water radiometry and lidar fluorosensor. Three empirical algorithms were used to estimate CHL from radiometric measurements: Ocean Chlorophyll 3 bands (OC3M_RAD_), Ocean Chlorophyll 4 bands (OC4v4_RAD_), and Ocean Chlorophyll 2 bands (OC2v4_RAD_). The satellite estimates of CHL were derived from data collected by the MODerate-resolution Imaging Spectroradiometer (MODIS) with a nominal 1.1 km resolution at nadir. Three algorithms were used to estimate chlorophyll concentrations from MODIS data: one empirical - OC3M_SAT_, and two semi-analytical - Garver, Siegel, Maritorena version 01 (GSM01_SAT_), and Carder_SAT_. In the present work, MODIS, lidar and *in situ* above-water radiometry and fluorometry are briefly described and the estimated values of chlorophyll retrieved by these techniques are compared. The chlorophyll concentration in the study area was in the range 0.01 to 0.2 mg/m^3^. In general, the empirical algorithms applied to the *in situ* radiometric and satellite data showed a tendency to overestimate CHL with a mean difference between estimated and measured values of as much as 0.17 mg/m^3^ (OC2v4_RAD_). The semi-analytical GSM01 algorithm applied to MODIS data performed better (*rmse* 0.28, *rmse-L* 0.08, *mean diff.* -0.01 mg/m^3^) than the Carder and the empirical OC3M algorithms (*rmse* 1.14 and 0.36, *rmse-L* 0.34 and 0.11, *mean diff*. 0.17 and 0.02 mg/m^3^, respectively). We find that *rmsd* values between MODIS relative to the *in situ* radiometric measurements are < 26%, *i.e.*, there is a trend towards overestimation of *R_RS_* by MODIS for the stations considered in this work. Other authors have already reported over and under estimation of MODIS remotely sensed reflectance due to several errors in the bio-optical algorithm performance, in the satellite sensor calibration, and in the atmospheric-correction algorithm.

## 1. Introduction

Ocean-color remote sensing has changed our perspective of ocean observation. Global maps of surface chlorophyll concentration (CHL), a proxy of phytoplankton primary productivity can be routinely produced [1, 2]. However, these satellite products are based on the application of generally complex bio-optical algorithms [3], including atmospheric correction models [4] applied on the radiance values measured by the remote sensors to estimate the water leaving radiances. In order to guarantee good quality and long term consistency of satellite data time series, orbital images must be calibrated and validated with the use of *in situ* measurements from research ships, moored buoys and drifters, for example [3].

Satellite images have been systematically used for monitoring the oceanic environment on the Brazilian Southeastern continental margin. However, only very seldom, has simultaneous *in situ* data been available in this region for the assessment of satellite algorithm accuracies. Aiming to advance scientifically and technically the current remote sensing data analysis procedures a research project - FITOSAT was jointly conducted by INPE, PETROBRAS R&D Center and other universities. One of the phases of this project involved an oceanographic cruise with the simultaneous acquisition of *in situ* and remote sensing data in the Campos Basin region. The simultaneous data acquisition enabled the meteo-oceanographic contextualization of the *in situ* data collection and allowed the evaluation of the remote sensing products.

Among other measurements, *in situ* estimates of CHL were obtained with laboratory fluorometry, above-water radiometry and through a lidar fluorosensor. The satellite estimates of CHL were derived from MODIS data. In this article, these different methods used for estimating CHL are briefly described and their results are statistically compared.

## 2. Data and Methods

### 2.1. In situ Chlorophyll

**Figure 1.**
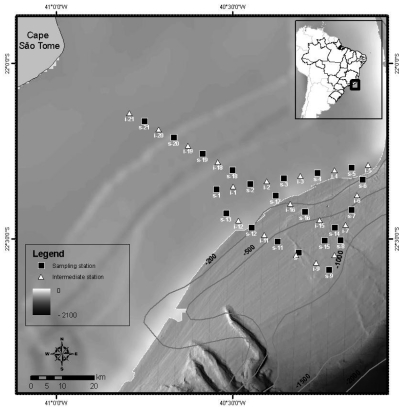
Study area and field stations occupied during November 2004 at the Brazilian Southeastern continental margin. Water sampling, above-water radiometry and lidar stations labeled with an “*s*” (black squares). Stations without water sampling labeled with an “*i*” (white triangles). Isobaths in meters.

Chlorophyll measurements were done at 18 stations during an oceanographic cruise held in November 2004. The study area included the Brazilian Southeastern continental shelf and slope region, from Cape Sao Tome (22°S) to Cape Frio (23°S), at Campos Basin, Rio de Janeiro (Figure 1). Phytoplankton abundance was estimated from 2 L surface water samples filtered through Millipore cellulose membranes (0.45 μm). The filters were kept in liquid nitrogen and the chlorophyll-*a* concentrations were determined after extraction in 90% acetone for 18 hours at 4°C in a Turner TD-700 fluorometer [5].

### 2.2. In situ Radiometry

Above water radiometric measurements of water leaving radiance and incident irradiance were obtained with a hyperspectral SPECTRON SE590 radiometer at 30 stations (Figure 1). Each radiance spectrum was sampled between 400–800 nm, with a 5 nm resolution. The protocol adopted for the measurements was the one proposed by Fougnie *et al.* [6], with a polarizer filter. The radiometric data were numerically integrated to simulate the spectral bands of SeaWiFS and MODIS orbital sensors, using the trapezoidal rule. The remote sensing reflectance, *R_RS_*(λ), was calculated by the following equation:

(1) $$R_{\text{RS}}=\frac{L_{w}(\lambda )}{E_{d}(\lambda )}$$

where *L_w_*(*λ*) is the water leaving spectral radiance and *E_d_* (λ) is the downwelling spectral irradiance incident on the sea surface. *E_d_* (λ) was estimated by the radiance reflected by a Spectralon plate [7], as follows:

(2) $$E_{d}(\lambda )=L(\lambda )f_{c}\pi$$

where *f_c_* is a correction factor estimated in the laboratory by the ratio of *L_ref_* (*λ*) of a standard reference (approximately 100%) by the Spectralon plate's *L*(*λ*) used in the field.

The SeaWiFS empirical algorithms Ocean Chlorophyll 4-bands - OC4v4 and Ocean Chlorophyll 2-bands - OC2v4, and the MODIS algorithm Ocean Chlorophyll 3-bands - OC3M were applied to the radiometric data to estimate CHL.

The OC2v4 algorithm estimates CHL based on a band ratio of *R_RS_*(490)/*R_RS_*(555) using a modified cubic polynomial function [3]:

(3) $$\text{CHL}=10,0^{(0,319-2,336R_{2S}+0,879R_{2S}^{2}-0,135R_{2S}^{3})-0,071}$$

where $R_{2S}=\log_{10}\left(R_{555}^{490}\right)$.

The algorithm OC4v4 also relates a band ratio with CHL using a polynomial function, but is based on the maximum band ratio determined as the highest ratio (*R_max_*) between the values of *R_RS_*(443)/*R_RS_*(555), *R_RS_*(490)/*R_RS_*(555), and *R_RS_*(510)/*R_RS_*(555) through the following function of forth order [3]:

(4) $$\text{CHL}=10,0^{\left(0,366-3,067R_{4S}+1,930R_{4S}^{2}-0,649R_{4S}^{3}-1,532R_{4S}^{4}\right)}$$

where *R*_4_*_M_* = log_10_(*R*_max_).

The algorithm OC3M also uses a polynomial function of forth order from *R*_max_ between *R_RS_(443)/R_RS_(550)* and *R_RS_(490)/R_RS_(550)*, through the following equation [3]:

(5) $$\text{CHL}=10,0^{\left(0,283-2,753R_{3M}+1,457R_{3M}^{2}-0,659R_{3M}^{3}-1,403R_{3M}^{4}\right)}$$

where *R*_3_*_S_* = log_10_(*R*_max_).

### 2.3. Lidar

The PUC-Rio fluorosensor lidar system operated in a pulse mode uses the excitation line at 532 nm of the doubled frequency radiation (second harmonic) of Q-switch Nd:YAG laser as light source. A rotating rectangular metal mirror directs the probing beam to the water surface and deflects the water backscattered signal to a 200 mm-diameter Newton-type reflecting telescope, which is filtered in order to eliminate elastic backscattering. This return signal passes through the entrance slit of the polychromator, which is assembled as an auto collimation lens focusing system. A 1024 elements array CCD camera with Peltier cooling system was used to detect the spectrum of the return signal.

Two types of radiation are collected: one is the water-Raman backscattering (which occurs at 655 nm) and the other is the fluorescence radiation generated when the light source interacts with the fluorescent elements present in the water. The laser light at this wavelength excites the fluorescence of chlorophyll, in a band centered at 685 nm, and of dissolved organic matter, in a region from 540 to 620 nm. Over 13.000 spectra were obtained during the campaign, with the lidar installed at the gangway of the *R/V Astro-Garoupa*. In order to validate each spectrum, an analysis of the Fourier Transform power spectrum was made, and then a validation criterion of these spectra was established. Of the total number of spectra collected during the vessel's course between the November 21st and 25th, 9,511 of them were the objects of analysis.

The intensities of the chlorophyll bands (*I_cl_*) were calculated according to the method developed by Barbosa [8] for the equipment. The values of *CHL* were obtained by means of an adjustment using calibration parameters obtained via laboratorial analysis of the samples collected during the present cruise and from a prior cruise at the same region. The calibration used is given by:

(6) $${\text{CHL}}_{\text{LIDAR}}=4,9*(I_{\text{cl}})-0,175$$

### 2.4. Remote Sensing

Ocean color remote sensing images acquired by MODIS sensor during the same period of the oceanographic cruise were processed as CHL fields with the application of one empirical and two semi-analytical algorithms. The MODIS sensor has 36 spectral bands, with eight dedicated to ocean color applications. These bands have a spatial nominal resolution of to 1.1 km and a temporal resolution of 1-2 days.

MODIS images were acquired locally by INPE's receiving station and processed using SeaDAS software distributed by NASA. Initially, the data were radiometrically calibrated to generate the water normalized upwelling radiances. The images considered of interest were selected in accordance to the study area. Atmospheric correction algorithms [4] were applied to each image using the more updated atmospheric ancillary files available before the calculation of CHL values.

As mentioned before, the OC3M empirical algorithm [3], and two semi-analytical algorithms, Garver, Siegel, Maritorena version 01 – GSM01 [9], and Carder [10] were applied to estimate CHL with MODIS data. Maritorena *et al.* [9] presented a protocol to improve the semi-analytical model initially proposed by Garver and Siegel [11], for global applications. The complete formulation of the model can be expressed as the following:

(7) $$L_{\text{WN}}(\lambda )=\frac{tF_{0}(\lambda )}{n_{w}^{2}}\sum_{i=1}^{2}g_{i}\left\{\frac{b_{bw}(\lambda )+b_{bp}(\lambda_{0}){\left(\lambda /\lambda_{0}\right)}^{-\eta }}{b_{bw}(\lambda )+b_{bp}(\lambda_{0}){\left(\lambda /\lambda_{0}\right)}^{-\eta }+a_{w}(\lambda )+Chl_{\text{ph}}^{*}(\lambda )+a_{\text{cdm}}(\lambda_{0})\exp[-S(\lambda -\lambda_{0})]}\right\}$$

where *L_WN_* is the normalized water leaving radiance, *t* is the air-sea transmission factor; *F_0_(λ)* is the extra-terrestrial solar irradiance; *n_w_* is the refraction index of water; *g_1_* = 0.0949 sr^-1^ and *g_2_* = 0.0794 sr^-1^; *bb_w_*(λ) is the backscattering of water; *a_w_*(λ) is the absorption by water; *bb_p_*(λ) is the backscattering by particles; $Chl_{\text{ph}}^{*}$ is the chlorophyll-*a* specific absorption coefficient; *S* is the spectral decay for the dissolved matter and detritus absorption (*cdm*); *η* is the exponent of the power law for the particulate backscattering coefficient; *λ_0_* is the wavelength 443 nm wavelength.

The Carder algorithm [10] utilizes a more complex approach. The components associated with the absorption of the pigments are divided from those associated with the degradation products (for example, *gelbstoff* and detritus, $a_{g}^{*}(\lambda )$). The absorption coefficient of phytoplankton chlorophyll, $a_{ph}^{*}(\lambda )$, is adjusted in relation to the chlorophyll concentration and the availability of light and nutrients. The distinction between the effects of the principal constituents is obtained by the spectral differences between $a_{ph}^{*}(\lambda )$ and $a_{g}^{*}(\lambda )$. Comparing sea surface temperature with nitrate depletion temperature (NDT) [12], the presence of big cells rich in chlorophyll and small cells poor in chlorophyll can be deduced from the satellite data [10]. The chlorophyll rich cells with low values of $a_{ph}^{*}(\lambda )$, i.e., with packed pigments, occurs generally in ambient with low level of light and rich in nutrients. On the other hand, chlorophyll poor cells but with high values of $a_{ph}^{*}(\lambda )$, i.e., without packaging, are present in ambient replete of photons, but poor in nutrients. During the development and validation of the Carder algorithm, *in situ* data sets were compartmented into 2 regions. In the first, the pigment packaging would not be expected, and a second one where this packaging effect would probably occur more frequently or more intensely. Besides, a global average algorithm was developed to be used in conditions where the packaging effect is unknown or transitional. The algorithm also alternates between the empirical and the semi-analytical formulation, using different coefficients for the varying levels of pigment packaging.

## 3. Results and Discussion

### 3.1. Statistical comparisons

The comparisons obtained between the *in situ* CHL and those estimated with MODIS data were calculated inside a time window of 12 hours. The pairs of data were composed between the *CHL_insitu_* and the median value of a 3 × 3 pixels (9 km^2^) centered on the geographical position of the sampling station in the equivalent MODIS image. The fluorometric data were statistically compared to the satellite estimates, above-water radiometric data and lidar data, through linear regression analysis, root mean square error (*rmse*), and transformed-rmse (*rmse-L*) [10].

*CHL_insitu_* values varied between 0.077 and 0.197 mg/m^3^ with a mean value of 0.12 (±0.04) mg/m^3^. These low values are typical of the oligotrophic waters of the Brazil Current (BC) as observed previously by other authors [13, 14]. With Z_90_ depths [15] of ∼13 m, the chlorophyll concentration was constant over one optical depth in 8 stations, with an increase in 10 stations. The mean chlorophyll concentration to a depth of one optical depth was 0.15 (±0.04) mg/m^3^, with a maximum value of 0.303 mg/m^3^.

**Figure 2.**
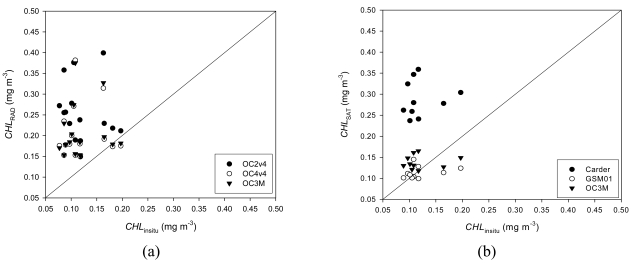
Scatter plots of chlorophyll concentration estimates obtained with (a) above-water radiometric data (three algorithms), and (b) MODIS ocean color remote sensing data (three algorithms) against *in situ* fluorometric estimates.

In general, the empirical algorithms applied to the *in situ* radiometric data overestimated *CHL_insitu_* (Figure 2). OC3M and OC4v4 presented a similar performance, with *rmse-L* (1.09, 1.08) and *rmse* (0.33, 0.32) as compared to OC2v4 (1.57 and 0.45), respectively (see Table 1). But the lower mean difference between estimated and measured values was calculated for OC4v4 (0.01 mg/m^3^).

In addition to the bio-optical algorithm performance, there may be some errors in the satellite sensor calibration and in the atmospheric-correction algorithm (see below).

Based on the MODIS chlorophyll fields, *in situ* data and numerical modeling was possible to observe an anticyclonic Brazil Current frontal eddy in the study area during the cruise [16]. Concurrent with the mesoscale eddy there was an uncommon sea floor oil seep event [17], which surface signature won't be discussed in detail here.

**Figure 3.**
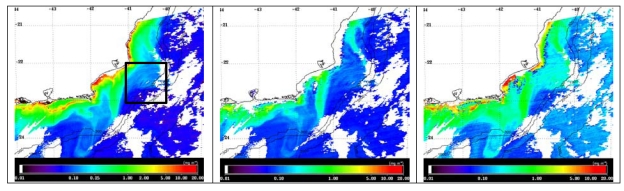
Surface distributions of chlorophyll concentration estimated with OC3M (left), GSM01 (center), and Carder (right) algorithms applied to MODIS data acquired on 11/25/2004 (see text for details). Isobaths in meters. Color table in logarithmic scale. Land and clouds are masked in white. Black square on the left indicates the *in situ* sampled area.

For illustration, examples of MODIS images processed using with the three algorithms tested in this study are shown (Figure 3). In the 11/25/2004 image, the oligotrophic waters of the BC are observed offshore, over the slope region, in dark blue colors (OC3M<0.08 mg/m^3^; GSM01<0.08 mg/m^3^; Carder<0.10 mg/m^3^). A surface signature of the mesoscale eddy with a mean diameter of 75-80 km was identified in front of Cape Sao Tome between 22.00°S–22.65°S, and 39.9°W–40.77°W, with relatively higher CHL values (0.10<OC3M<0.15 mg/m^3^; 0.08<GSM01<0.13 mg/m^3^; 0.20<Carder<0.30 mg/m^3^).

**Figure 4.**
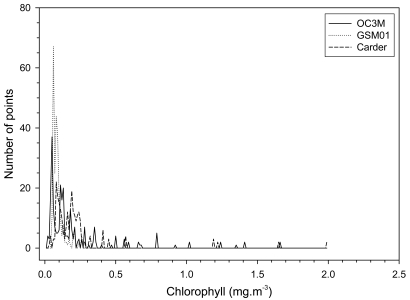
Histograms of MODIS chlorophyll concentration estimates obtained with OC3M, GSM01, and Carder algorithms for the *in situ* sampled area on 11/25/2004 (see text for details).

In order to perform a quantitative analysis of differences between the images produced by the three algorithms, we compared the histograms of the full area (not shown) and of the *in situ* sampled area (Figure 4, Table 2). Considering the full area, the CHL values varied similarly (0.011–19.929 mg/m^3^ OC3M, 0.010–19.862 mg/m^3^ Carder, 0.010–19.154 mg/m^3^ GSM01). But the mean value of the GSM01 (0.15 ± 0.25 mg/m^3^) was relatively lower than OC3M (0.45 ± 1.26 mg/m^3^) and Carder (0.43 ± 1.07 mg/m^3^). Now considering the *in situ* sampled area (Figure 4) the relative pattern was the same (Table 2) with the GSM01 mean value (0.10 ± 0.07 mg/m^3^) relatively lower than OC3M (0.21 ± 0.27 mg/m^3^) and Carder (0.23 ± 0.27 mg/m^3^).

Ocean color satellite algorithms OC3M and Carder overestimated on average the values of *CHL_insitu_*, with a mean difference of 0.02 and 0.17 mg/m^3^, respectively (Table 1), but the algorithm GSM01 has underestimated the relatively higher *in situ* concentrations (Figure 2). Statistically, the best performance was obtained with the semi-analytical algorithm GSM01, with the lowest values of *rmse-L* (0.28), *rmse* (0.08), and mean difference (-0.01 mg/m^3^), respectively. The empirical algorithm OC3M also presented a good performance, with *rmse-L* equal to 0.36, *rmse* of 0.11, and mean difference of 0.02 mg/m^3^ (see Table 1).

There are some discrepancies between the datasets, which may be due to different reasons. A relevant aspect is that there is usually a time difference between the satellite and *in situ* measurements. This could be particularly significant when the chlorophyll distribution features are dynamic, as during the cruise period when an anticyclonic eddy was active in the study area. Besides, to evaluate semi-analytical algorithms properly, inherent optical properties measurements are required, but these measurements were not acquired for this work. The absorption coefficient is one of the inherent optical properties that affect the reflectance of the aquatic systems. Therefore, the knowledge about the total absorption and its components is fundamental to improve the description of the spectral reflectance variability and chlorophyll concentration estimated by ocean color remote sensing sensors [18].

**Figure 5.**
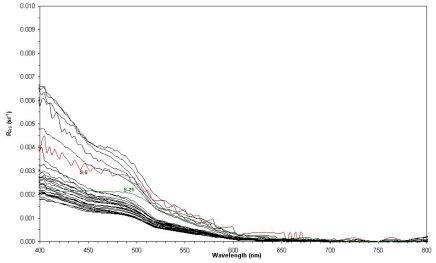
Surface remote sensing reflectance spectra obtained by above-water radiometry during the cruise FITOSAT I cruise, November 2004, in the Brazilian Southeastern continental shelf and slope. Stations *S-5* and *S-21* in red and green, respectively (see text).

The spectral distributions of *R_RS_* measured at 30 stations are shown on Figure 5. In general, all the spectra are typical of Case 1 oceanic and oligotrophic waters with low chlorophyll concentrations. The noise observed at station *S-5* (red on Figure 5) was due to the low light level at the time of data acquisition. The different shape of the spectrum at station *S-21* corresponds to more coastal waters (green on Figure 5), with relatively higher chlorophyll concentration (0.54 mg/m^3^ OC3M) and with the probable presence of other optically active components.

**Figure 6.**
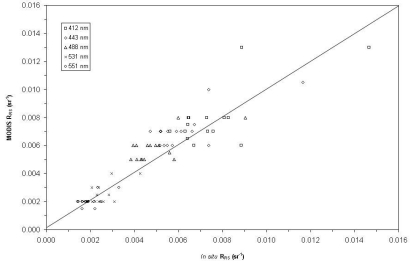
Scatter plot of MODIS remote sensing reflectance (*R_RS_*) with respect to *in situ* above-water *R_RS_* for 15 stations where inter-comparison were made.

A spectral comparison between average MODIS-derived *R_RS_* and the *in situ* estimates from the radiometer for 15 “match-up” stations is shown in Figure 6. The 1:1 line of perfect correspondence is also shown. We define *rmsd* at any wavelength *λ* as [19]:

(8) $$\text{rmsd}(\lambda )=100\sqrt{\left[N^{-1}\sum \frac{{\left(R_{\text{RS}}^{\text{SAT}}-R_{\text{RS}}^{\text{RAD}}\right)}^{2}}{\left(R_{\text{RS}}^{\text{RAD}}\right)}\right]},$$

where $R_{RS}^{SAT}$ and $R_{\text{RS}}^{\text{RAD}}$ are the remote sensing reflectance estimates from MODIS and the above-water radiometer, respectively at the same station and *N* is the number of matched stations, where the summation Σ is over *N* stations (here *N* = 15). We find that *rmsd* values between MODIS relative to the *in situ* radiometric measurements are 25.3% at 412 nm, 25.9% at 443 nm, 25.9% at 488 nm, 15.6 % at 531 nm, and 13.2% at 551 nm, i.e., largest for 443–448 nm, and least for 551 nm.

It was shown that *R_RS_* measured *R_RS_* and estimated from MODIS are generally in agreement. The OC3M empirical algorithm overestimated *CHL_insitu_* when applied to measured *R_RS_* and to satellite-derived *R_RS_*. However, OC3M bio-optical algorithm performed much better with MODIS-derived *R_RS_* (see Table 1). The reduced number of satellite “match-ups” available for analysis (N = 10) compared to the *in situ* radiometric measurements (N = 17) may partially account for the difference found. Furthermore, as mentioned before, there are some discrepancies between the datasets, which may be due to different reasons. The vertical chlorophyll profile was homogeneous over one optical depth only in 8 stations, with concentration increasing in other 10 stations. And the “match-up” stations are not coincident. In addition, we speculate here that compensation could occur between errors in atmospheric correction, satellite sensor calibration and uncertainty in the bio-optical algorithm.

**Figure 7.**
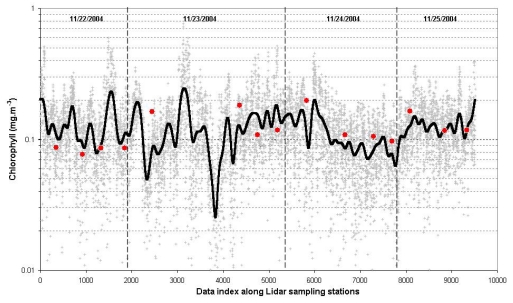
Surface chlorophyll concentration estimated with the along-track lidar sampling during the cruise FITOSAT I cruise, November 2004, in the Brazilian Southeastern continental shelf and slope compared with fluorometric estimates (grey crosses – Lidar measurements; black line – Lidar processed; red circles – fluorometric).

The fluorescence spectra processed from the lidar data are presented on Figure 7. The chlorophyll concentrations estimated with the lidar (*CHL_LIDAR_*) at the stations with simultaneous water sampling for fluorometric analysis varied between 0.064 and 0.016 mg/m^3^, with a mean value of 0.12 (±0.03)mg/m^3^, very similar to *CHL_insitu_*. In fact, a paired t-test confirmed that the two mean values are statistically equal. The performance of the lidar was similar to that obtained by the *in situ* fluorometric and radiometric methods, with *rmse, rmse-L* and *mena diff*. values of 0.14, 0.48, and 0.01 mg/m^3^ respectively.

The amplitude of laser excited fluorescence on Figure 7, where the observations were conducted at the same point, can be associated with a diurnal variation. The laser excited fluorescence exhibits a high fluorescence in the night while phytoplankton is not conducting photosynthesis. During the day, exhibited a low fluorescence while phytoplankton is already excited by the solar irradiation and emitting less fluorescence against the laser excitation. The relationship between phytoplankton biomass and pigment content varies widely with light acclimation and nutrient status, while the fluorescence yield also varies with the physiological condition of the phytoplankton and with the type of plankton present. In both laboratory studies and in the field, researchers have observed a daily rhythm of fluorescence that is not correlated with diel changes in the concentration of chlorophyll-*a*. During periods of high irradiance fluorescence tends to be lower than it is at night [20–22].

We learned from previous experiments that our lidar measurements are well correlated with the surface (∼1 m) chlorophyll concentration. The analysis conducted to adjust the lidar with the fluorometric estimates was based on the observation that de relative standard deviation (variance coefficient) of the latter was much higher than the former. As mentioned on Section 2.3, the values of *CHL_LIDAR_* were obtained by means of a single equation (see Equation 6).

Spectrally resolved local and remote laser induced fluorescence detectors are emerging as very reliable tools for the collection of information on fluorescent targets for sea and land diagnostics purposes [23, 24]. Barbini *et al.* [25] also compared lidar data and local spectrophotometer and/or spectro-fluorometer measurements in Antarctica. The observed discrepancies were explained as a result of the fact that lidar signals were collected from a layer where mixing processes can take place, whereas seawater samples were collected at a well defined depth.

Barbini *et al.* [26] compared lidar measurements of surface chlorophyll-*a* concentrations in the Ross Sea and in the Antarctica-New Zealand transect to data collected by SeaWiFS. The authors concluded that in coastal waters, lidar values were significantly higher than the orbital estimates. In open waters the differences were smaller and the sampling stations were considered robust to calibrate the SeaWiFS algorithm against the lidar measurements in the Ross Sea.

## 4. Conclusions

To our best knowledge this is the first published result of simultaneous measurements of surface chlorophyll concentration in the Brazilian Southeastern continental shelf and slope region using *in situ* fluorometric, above-water radiometry, and lidar, complemented with ocean color remote sensing MODIS imagery.

The empirical algorithms applied to the *in situ* radiometric and satellite data showed a tendency of overestimating the *in situ* CHL values by as much as 0.17 mg/m^3^ (for OC2v4RAD). The semi-analytical GSM01 algorithm applied to MODIS data presented the best performance (*rmse* 0.28, *rmse-L* 0.08, *mean diff*. -0.01 mg/m^3^). With the available dataset, MODIS showed a trend in overestimating the *R_RS_* (up to 26%) relative to the *in situ* measurements.

When a comprehensive bio-optical dataset from future cruises is collected, a regional algorithm can be developed, to suit local Brazilian Southeastern continental shelf and slope waters. However, this new algorithm must be able to estimate not only CHL, but also other optically active constituents to allow the monitoring of other important water quality parameters and to advance in the ecosystemic study of the interest area.

A better calibration of the lidar equipment is necessary to account for the daily rhythm of fluorescence that is not correlated with diel changes in CHL. This would provide the opportunity to have a CHL onboard monitoring equipment that would automatically and autonomously determine the surface chlorophyll concentrations along the ship track.

## Figures and Tables

**Table 1.** Comparison between different *in situ* and satellite estimates of surface chlorophyll-*a* concentration in the Brazilian Southeastern continental shelf and slope waters in November 2004. (N= number of observations; RAD= *in situ* radiometry; SAT= MODIS data).

**Algorithm/Lidar**	***rmse-L***	***Rmse***	**N**	mean diff.*
OC2v4_RAD_	1.57	0.45	17	0.17
OC4v4_RAD_	1.08	0.32	17	0.01
OC3M_RAD_	1.09	0.33	17	0.06
OC3M_SAT_	0.36	0.11	10	0.02
GSM01_SAT_	0.28	0.08	10	-0.01
Carder_SAT_	1.14	0.34	10	0.17
CHL_LIDAR_	0.52	0.16	15	0.01

* (estimated – measured)

**Table 2.** Comparison between histograms of MODIS chlorophyll concentration estimates obtained with 3 algorithms for the full image area and for the *in situ* sampled area (mg/m^3^).

**Algorithm**	***Min.***	***Max.***	***Mean***	***std.***
Full area				
OC3M	0.01	19.93	0.45	1.26
GSM01	0.01	19.15	0.15	0.25
Carder	0.01	19.86	0.43	1.07

Sampled area				
OC3M	0.02	1.66	0.21	0.27
GSM01	0.05	0.26	0.10	0.04
Carder	0.03	2.00	0.23	0.27
